# Delayed pancytopenia following ingestion of wild mushrooms consistent with *Hypholoma fasciculare*: A case report

**DOI:** 10.1097/MD.0000000000051400

**Published:** 2026-11-06

**Authors:** Hyeonbeom Byeon, Juho An

**Affiliations:** a Department of Emergency Medicine, Ajou University School of Medicine, Suwon, Republic of Korea.

**Keywords:** bone marrow suppression, case report, *Hypholoma fasciculare*, mushroom poisoning, pancytopenia

## Abstract

**Rationale:**

*Hypholoma fasciculare* (sulfur tuft) is a toxic mushroom commonly associated with gastrointestinal symptoms. However, hematologic toxicity related to *Hypholoma* species has rarely been reported. In particular, delayed-onset pancytopenia following mushroom ingestion remains poorly understood.

**Patient concerns:**

A 57-year-old man presented to the emergency department after ingesting self-collected wild mushrooms. Approximately 10 days after ingestion, he developed fever and generalized weakness.

**Diagnoses:**

Laboratory evaluation revealed delayed-onset pancytopenia. Extensive infectious, autoimmune, and hematological investigations, including viral serology and bone marrow examinations, excluded primary hematological malignancies, aplastic anemia, and infectious causes. Based on the clinical course and the exclusion of alternative etiologies, toxin-mediated bone marrow suppression following mushroom ingestion was considered the most plausible diagnosis.

**Interventions:**

The patient was conservatively managed with close monitoring and supportive care. No specific antidotal or immunosuppressive agents were administered.

**Outcomes:**

Hematological parameters gradually recovered without targeted interventions. Follow-up laboratory tests demonstrated complete resolution of the pancytopenia, and the patient remained clinically stable without recurrence during outpatient follow-up.

**Lessons:**

This case highlights delayed pancytopenia as a potential manifestation of toxic mushroom ingestion, even in the absence of severe initial symptoms. Recognition of this pattern may facilitate a timely diagnosis and appropriate monitoring.

## 1. Introduction

Ingestion of wild mushrooms remains a common cause of toxicity worldwide, with clinical manifestations ranging from mild gastrointestinal symptoms to life-threatening organ failure.^[1-4]^ The toxic effects of mushroom ingestion are typically classified based on the latency of symptom onset and the primary target organs such as the liver, kidneys, and nervous system. In contrast, hematologic complications following mushroom poisoning are uncommon and poorly characterized, particularly when they are delayed.^[2–4]^

*Hypholoma fasciculare*, commonly known as sulfur tuft, is a widely distributed toxic mushroom that grows in clusters on decaying wood. The ingestion of *Hypholoma* species has been primarily associated with gastrointestinal toxicity, and severe systemic complications have rarely been reported.^[5-7]^ Although several toxic compounds, including fasciculols and fasciculic acids, have been isolated from *H fasciculare*, their clinical effects in humans remain unclear.^[5,8-11]^ To our knowledge, delayed hematologic toxicity following ingestion of mushrooms morphologically consistent with *H fasciculare* has not been well documented in the literature.

Herein, we report a case of delayed-onset pancytopenia following ingestion of wild mushrooms morphologically consistent with *H fasciculare* based on the patient’s exposure history and photographic documentation without mycological or molecular confirmation.

This case highlights toxin-mediated transient bone marrow suppression as a potential but under-recognized manifestation of mushroom poisoning and underscores the importance of considering delayed hematologic toxicity in patients with a history of wild mushroom ingestion.

## 2. Case report

A 57-year-old man with a history of hypertension was referred to our tertiary care center for evaluation of persistent pancytopenia. Approximately 2 weeks before the present episode, from September 14 to 18, 2025, he was admitted to a local hospital after ingesting a small amount of wild mushrooms that were clinically suspected to be *Podostroma cornu-damae*. He remained clinically stable throughout hospitalization, and laboratory investigations revealed no hematologic abnormalities. He was discharged after a short period of observation. This previous hospitalization represented a separate clinical episode and was unrelated to the present mushroom ingestion described in this report. On September 29, 2025, he consumed 4 to 5 newly collected wild mushrooms from a mountain that were subsequently considered morphologically consistent with *H fasciculare* based on photographic documentation and exposure history, although mycological or molecular confirmation was not available.

He remained asymptomatic immediately after ingestion but developed diarrhea, abdominal pain, and fever on October 3, 2025. He was admitted to the referring hospital on October 4, 2025, where supportive treatment was initiated. During hospitalization, serial laboratory examinations demonstrated persistent pancytopenia involving all 3 hematopoietic cell lines. Because the cytopenia persisted without an identifiable cause, he was transferred to our institution on October 9, 2025, for further evaluation and management.

On presentation, the patient was alert and hemodynamically stable. Vital signs revealed fever and tachycardia, but no hypotension or respiratory compromise. Physical examination revealed no lymphadenopathy, hepatosplenomegaly, skin rashes, or bleeding. Initial laboratory evaluation demonstrated severe pancytopenia, with white blood cell (WBC) count: 0.9 × 10^3^/µL, absolute neutrophil count: 171/µL, hemoglobin: 8.9 g/dL, and platelet count: 51 × 10^3^/µL. Red blood cell indices were within the normal ranges. Inflammatory markers were elevated, whereas liver enzymes, renal function, creatine kinase, and cardiac biomarkers were within normal limits. Coagulation studies revealed mild abnormalities without any clinical evidence of bleeding. During the early hospital course, serial complete blood counts (CBCs) revealed further deterioration, reaching nadir values: WBC count: 0.5 × 10^3^/µL, absolute neutrophil count: 5/µL, hemoglobin: 7.7 g/dL, and platelet count: 19 × 10^3^/µL. A peripheral blood smear revealed no blasts or dysplastic features.

An extensive diagnostic evaluation was performed to identify the potential causes of pancytopenia. Tests for respiratory viral infections, including influenza viruses, respiratory syncytial virus, adenovirus, human metapneumovirus, parainfluenza virus, and SARS-CoV-2, were negative. The severe fever with thrombocytopenia syndrome virus test was negative. Autoimmune and vasculitic etiologies, including negative antineutrophil cytoplasmic antibodies, anti–glomerular basement membrane antibodies, and rheumatoid factor, were excluded. There was no laboratory evidence of hemolysis or disseminated intravascular coagulation. Bone marrow aspiration and biopsy revealed hypocellular marrow with an overall cellularity of approximately 15% to 30%. The erythroid lineage was markedly decreased, and the myeloid series showed mild hypoplasia with preserved maturation. While megakaryocyte counts were normal, plasma cell counts increased with no evidence of monoclonality. No hemophagocytosis was observed. Cytogenetic analysis revealed a normal male karyotype, without structural chromosomal abnormalities. Overall, these findings are consistent with transient bone marrow suppression. The serum ferritin level was markedly elevated, reaching a peak level of 2669 ng/mL, but gradually decreased during the recovery phase. Despite hyperferritinemia, no clinical, laboratory, or histopathological features were suggestive of hemophagocytic lymphohistiocytosis.

The patient was conservatively managed with close monitoring and supportive care. No specific antidotal therapy, immunosuppressive treatment, hematopoietic growth factor therapy, or blood transfusions were administered. Hematological parameters gradually improved over time, with recovery of white blood cell and platelet counts.

At outpatient follow-up, CBCs demonstrated sustained normalization of all hematological parameters. The patient remained clinically well, without recurrence. The temporal changes in hematologic parameters are summarized in Table 1, and representative photographs of the wild mushrooms ingested by the patient are shown in Figure 1.

**Table 1:** Time course of hematologic parameters following wild mushroom ingestion.

Time point	WBC (×10^3^/µL)	ANC (/µL)	Hemoglobin (g/dL)	Platelet count (×10^3^/µL)	Clinical notes
Mushroom ingestion (September 29, 2025)	–	–	–	–	Ingestion of multiple self-collected wild mushrooms
Symptom onset (October 3, 2025)	–	–	–	–	Development of diarrhea, abdominal pain, and fever
Initial presentation to our hospital (October 9, 2025)	0.9	171	8.9	51	Severe pancytopenia identified
Hematologic nadir (October 10, 2025)	0.5	5	7.7	19	Lowest recorded hematologic values
Recovery phase (October 20, 2025)	4.5	>1000	9.5*	237	Spontaneous hematologic recovery without specific therapy
Outpatient follow-up (October 28, 2025)	6.1	3477	11.2	506	Sustained hematologic recovery

ANC = absolute neutrophil count, WBC = white blood cell count.

Reference ranges: WBC, 4.0–10.0 × 10^3^/µL; ANC, ≥1500/µL; hemoglobin, 13.0–17.0 g/dL (adult male); platelet count, 150–400 × 10^3^/µL.

* Hemoglobin level during the early recovery phase.

**Figure 1.**
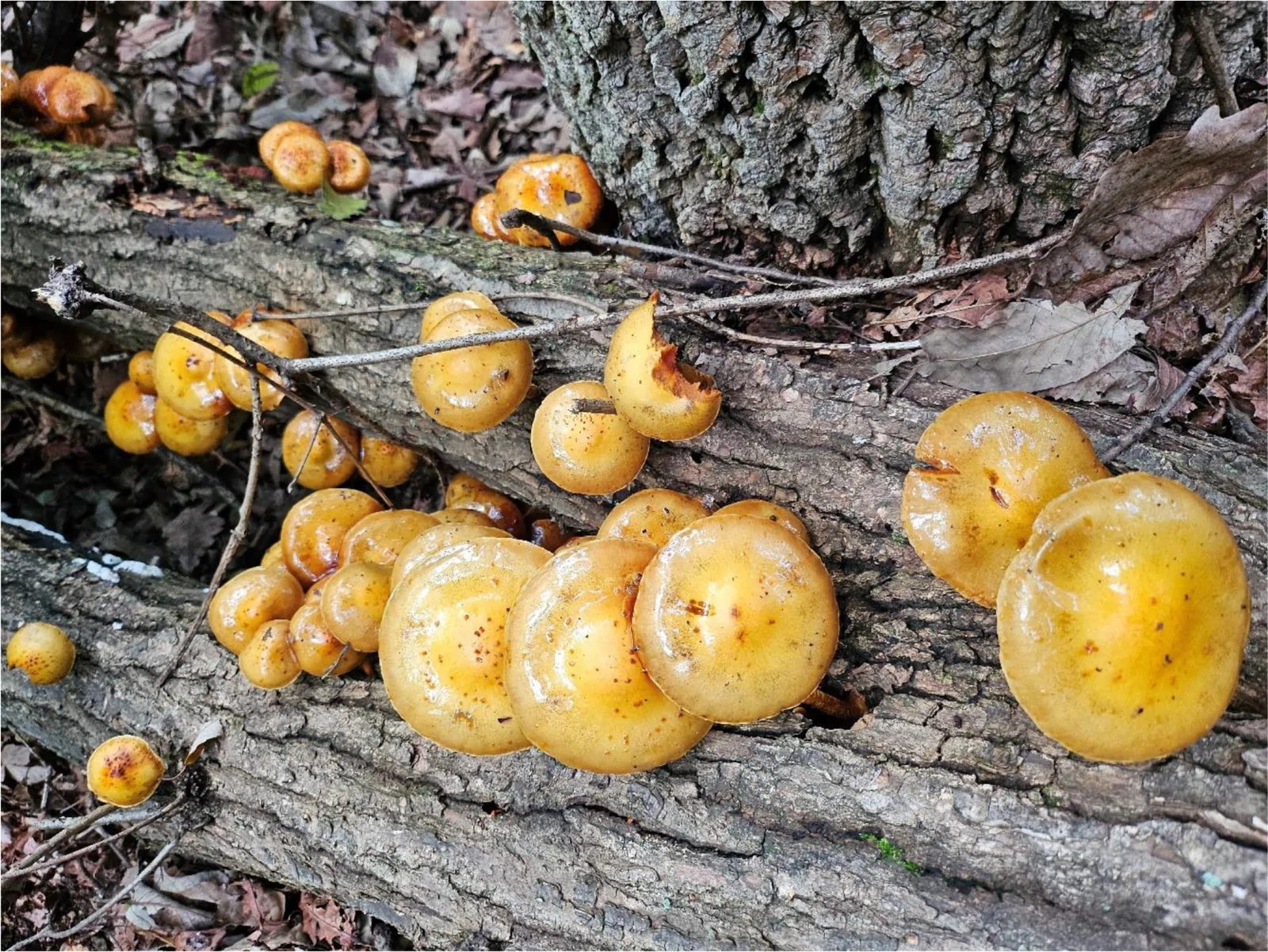
Photographic documentation of ingested wild mushrooms. Photographs of wild mushrooms collected and ingested by the patient showed clustered growth on decaying wood with yellowish to orange caps. The macroscopic appearance was most consistent with that of *Hypholoma fasciculare* (sulfur tuft). Species-level identification was based on photographic documentation and patient history without mycological or molecular confirmation.

## 3. Discussion

This case describes delayed-onset pancytopenia following the ingestion of wild mushrooms, most consistent with *H fasciculare*. The clinical course was characterized by an initial asymptomatic period, subsequent development of severe pancytopenia involving all 3 hematopoietic lineages, and spontaneous recovery without specific intervention. This temporal pattern strongly suggests a toxin-mediated transient suppression of bone marrow function.

Mushroom poisoning is classically categorized based on the latency of symptom onset and the primary target organ affected.^[2]^ While early-onset gastrointestinal toxicity is well-recognized, delayed systemic complications are less common and often pose diagnostic challenges.^[2,4]^ Hematologic manifestations after mushroom ingestion are particularly rare and have been most frequently reported in association with trichothecene-containing species, such as *Podostroma cornu-damae*, which cause profound bone marrow failure.^[12]^ In contrast, *H fasciculare* has been primarily associated with gastrointestinal toxicity, and hematological involvement has rarely been documented.^[6,7]^

In the present case, extensive evaluation excluded alternative causes of pancytopenia, such as infectious, autoimmune, and primary hematological disorders. Bone marrow examination revealed hypocellularity with relative preservation of megakaryocytes, no dysplasia, and no evidence of malignancy or hemophagocytosis. These findings, together with the self-limiting clinical course and complete hematological recovery, were consistent with transient bone marrow suppression rather than irreversible marrow failure. Importantly, no immunosuppressive therapy or hematopoietic growth factors were required, further supporting a reversible mechanism of toxicity.

Several toxic compounds have been isolated from *H fasciculare*, including fasciculols and fasciculic acids, which have been shown to interfere with intracellular signaling pathways, such as calmodulin-mediated processes.^[5,8-11]^ Although direct evidence linking these compounds to human bone marrow toxicity is lacking, it is biologically plausible that exposure to these toxins can impair hematopoietic cell proliferation or survival transiently. The delayed onset observed in this case may reflect toxin metabolism, cumulative exposure, or indirect effects on the marrow microenvironment, rather than immediate cytotoxicity.

This case highlights the importance of considering delayed hematological complications in patients with a history of wild mushroom ingestion, even when the initial symptoms are mild or nonspecific. Serial monitoring of CBCs is warranted in selected patients, particularly when exposure history is uncertain or involves unfamiliar mushroom species. The recognition of this pattern may prevent unnecessary invasive interventions and facilitate appropriate supportive management.

This study has some limitations. Species-level identification was based on the patient’s history and photographic documentation without mycological or molecular confirmation; therefore, the diagnosis should be interpreted as morphologically consistent with *H fasciculare*. Additionally, multiple wild mushrooms were ingested, raising the possibility of mixed exposure. Nevertheless, the consistent temporal relationship between ingestion and hematological deterioration, along with the exclusion of alternative etiologies and the complete recovery, supports the toxin-mediated process as the most plausible explanation.

## 4. Conclusion

In conclusion, this case expands the clinical spectrum of mushroom-related toxicity by demonstrating delayed, reversible pancytopenia due to transient bone marrow suppression following the ingestion of wild mushrooms, most consistent with *H fasciculare*. Even in the absence of severe initial symptoms, clinicians should be vigilant of delayed hematological complications following exposure to potentially toxic mushrooms. Careful history-taking, exclusion of alternative etiologies, and serial monitoring of hematological parameters are essential for timely diagnosis and appropriate management. Awareness of delayed hematological toxicity after mushroom ingestion is critical for proper follow-up and patient safety.

## Author contributions

**Conceptualization:** Hyeonbeom Byeon.

**Data curation:** Hyeonbeom Byeon.

**Supervision:** Juho An.

**Writing – original draft:** Hyeonbeom Byeon.

**Writing – review & editing:** Juho An.
